# Offspring of obese mice display enhanced intake and sensitivity for palatable stimuli, with altered expression of taste signaling elements

**DOI:** 10.1038/s41598-020-68216-7

**Published:** 2020-07-29

**Authors:** Ezen Choo, Lauren Wong, Patricia Chau, Jennifer Bushnell, Robin Dando

**Affiliations:** 1 Biomedical and Biological Sciences, College of Veterinary Medicine, Cornell University, Ithaca, NY 14853 USA; 2 College of Arts and Sciences, Cornell University, Ithaca, NY 14853 USA; 3 Division of Nutritional Sciences, Cornell University, Ithaca, NY 14853 USA; 4 College of Engineering, Cornell University, Ithaca, NY 14853 USA; 5 Department of Food Science, Cornell University, Ithaca, NY 14853 USA

**Keywords:** Metabolism, Obesity

## Abstract

Maternal body mass index and gestational weight gain predict future obesity status of the offspring. In studies of both rodents and non-human primates, maternal obesity also predicts a preference for palatable foods in the offspring. In this study, we used C57BL/6J mice to investigate whether an underlying cause for an increase in palatable food consumption in the offspring of obese mice was a change in taste function. Adult female mice were fed a normal chow (NC) or a high fat diet (HFD) for 5 weeks before mating, then also during the gestation (3 weeks) and lactation (3 weeks) periods, with offspring always maintained on a normal chow diet; thus the only experience offspring had with high fat food was via maternal exposure. Offspring exhibited similar weight, blood glucose levels and baseline water and chow intake in adulthood. Taste response was assessed after reaching maturity, using brief-access taste testing, with female offspring of obese dams showing an enhanced response to sucrose, and both sexes consuming more sucrose, sucralose and high fat diet if from obese mothers. Offspring also exhibited increased taste bud expression of mRNA for sweet receptor subunits T1R (Taste receptor type) 2 and 3, as well as other markers associated with taste signaling. Taste morphology in both groups appeared similar. Results indicate that obesity in the mother may lead to unhealthy feeding behavior in the offspring, correlating with altered expression of taste signaling elements, which likely drive increased avidity for palatable foods.

## Introduction

### Obesity and metabolic programming

Obesity is a chronic worldwide health issue with profound associated healthcare implications. Obesity is increasing at a higher rate in women than men^1^, and is also increasing in children^2^. In the United States, half women of childbearing age are overweight or obese^3^. Maternal obesity puts a child at increased risk for both childhood and adulthood obesity^4^, thus future generations may be at risk for obesity even before birth. Events occurring in utero can have long-term influences on disease risk later in life, termed ‘early life programming’, or the fetal origins hypothesis^5,6^. Maternal obesity and over-nutrition are now recognized as programming factors, leading to permanent changes in offspring metabolism, behavior, and appetite regulation, and a propensity for developing obesity, metabolic, and behavioral problems^7–9^. Recently, links have been uncovered between obesity and the taste system, with studies in mice^10,11^ and humans^11,12^ suggesting weight gain may influence the taste system.

### Maternal adiposity predisposes offspring to diet-induced obesity

Rodent studies of maternal obesity demonstrate that the offspring, when challenged with HFD after weaning, are predisposed to weight gain, poor glycemic control, and metabolic dysregulation^13,14^. This propensity for diet-induced obesity in the offspring of obese dams has been linked with alterations to the reward system and the hypothalamus^15^, suggesting that regulation of reward-related feeding is affected in such a model^16^. Thus, maternal obesity may contribute to diet induced obesity in the offspring, when exposed to a highly palatable diet as adults^17^.

Pregnancy in humans is a period marked by altered food intake, possibly due to changes in taste function while pregnant^18^. When mice are presented with hyper-palatable foods at weaning, the offspring of HFD-fed dams overconsume high-fat and high-sugar foods, and become obese sooner than offspring of dams maintained on a control diet^19^. When offspring are studied in adulthood, while there is no initial difference in body weight, differences between groups emerge when the animals are presented with a palatable diet^20,21^. These same (female) offspring also have higher preferences for corn oil than controls, suggesting weight gain in offspring of obese dams may be due in part to changes in taste. Elevated consumption levels are also associated with increased preference for palatable foods^22,23^. Such preferences for fat and sweet are positively correlated with overweight and obese status in adolescent humans^24^, and in nonhuman primates (*Macaca fuscata)*, dams fed HFD produce offspring that overconsume fat and sucrose relative to the controls^25^.

### Perinatal flavor programming

In neonates, sweet, umami, and low concentrations of salty substances are preferred, whereas bitter and sour substances alone are typically rejected. Studies show that exposure to certain taste stimuli during infancy or early childhood can modify these seemingly innate tendencies, and alter dietary preferences in children^26–31^. For instance, studies by Mennella et al.^32^ reveal that mothers fed carrot juice during their third trimester pass carrot flavor acceptance to their offspring. There is evidence linking the parental diet and nutritional status to multiple phenotypic traits in the offspring^33^, highlighting the perinatal period as a window for nutritional intervention that can have lifelong effects on health and dietary preference in the offspring. The present study was designed to examine the effects of maternal obesity on the taste bud, and on resultant taste behavior in the offspring of obese mice. We hypothesized that maternal obesity would increase behavioral responses to palatable foods via the modulation of taste transmission elements in the offspring, highlighting the taste bud as a locus for juvenile obesity.

## Methods

### Animals

**Figure 1:**
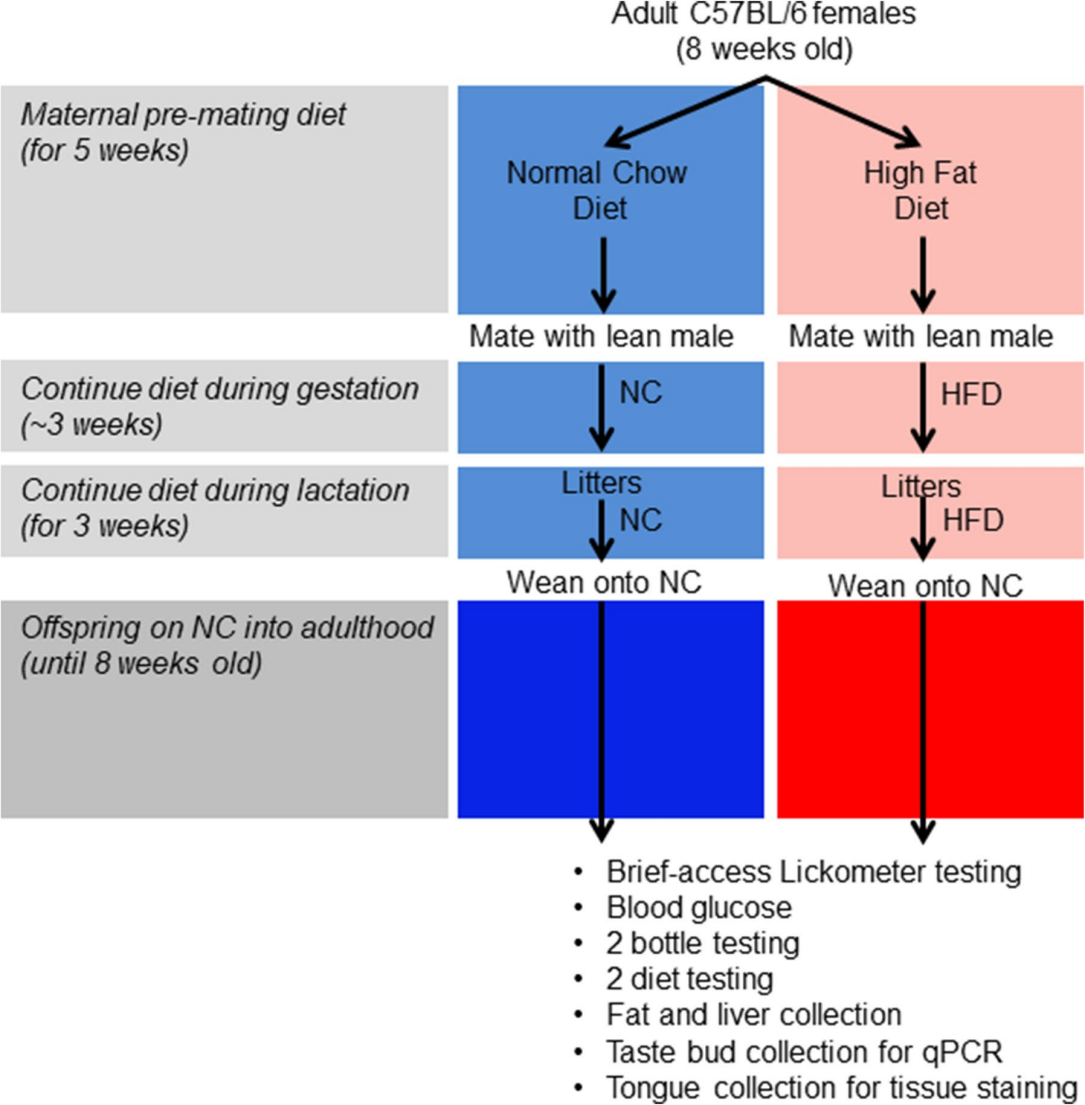
Schematic of study design. 8 week old females were fed normal chow (NC) or high-fat diet (HFD) for 5 weeks pre-conception and throughout the gestation/ lactation period. All offspring were then weaned onto regular chow and examined as adults, at 8 weeks of age giving them time to reach maturity.

All procedures were reviewed and approved by the Institutional Animal Care and Use Committee at Cornell University. Experiments were performed in accordance with relevant guidelines and regulations. In-house bred C57BL/6J female mice were assigned to one of two diets (see Supplemental Table 1) ad libitum at 8 weeks of age: normal chow (NC, 18% kcal from fat) or a high-fat diet (HFD, 58.4% kcal from fat). Females were maintained on their respective diets during the pre-mating period (8–13 weeks of age). After 5 weeks on respective diets, females were mated with healthy lean males maintained on NC. Day 1 of pregnancy was determined by detection of a copulatory plug. Pups were weaned at 3 weeks onto NC. At 7 weeks offspring were single housed and given a week to acclimatize before testing (Fig. 1). Additional groups of females were also placed on HFD only before mating (but not through pregnancy or lactation), or only after mating. Full composition of diets can be found at Ferramosca et al.^34^.

### Baseline measurement

At 8 weeks of age offspring were weighed and baseline measurements of water and NC intake were recorded over 48 h. Before behavioral testing, mice were fasted for 4 h, and circulating blood glucose levels from tail blood was measured using a OneTouch UltraMini glucose meter (OneTouch, Systems, Sunnyvale, CA).

### Brief access lickometry

Taste responses were measured using a brief-access Davis Lickometer (DiLog Instruments, Tallahassee, FL). Training and testing schedule was adapted from Glendinning et al.^35^. Before testing, mice were partially water and food restricted by providing 1 mL of water and 1 g of normal chow for 23.5 h. Once the mouse initiated licking on the presented bottle, the timer started for 5 s and then the shutter closed. Each test session lasted no more than 1 h in total, during which the mouse could initiate up to 5 blocks of 7 concentrations (i.e. 35 total presentations). Lick responses were normalized, fit to nonlinear variable slope concentration–response curves, and compared using extra sum-of-squares F test. Tastant concentration–lick ratio response curves were fitted to the mean data for each group using a classical four parameter logistic sigmoidal dose–response equation in the nonlinear regression suite of GraphPad Prism (v5.0, GraphPad Software, San Diego, CA).

### Two bottle testing – sucrose and sucralose

Mice were tested with water and sucrose (0.001, 0.02, or 0.1 M) or sucralose (0.1, 0.3, or 1.0 mM). All solutions were prepared fresh, purchased from Sigma Aldrich, St Louis, MO, unless stated. Mice were given simultaneous access to the two bottles with consumption measured over 48 h. Bottle placement was random and switched after 24 h.

### Diet preference and intake

Diet preference testing methods were adapted from Vucetic et al.^36^ and Carlin et al.^37^. Mice were single housed and trained to consume NC from dual hoppers for 48 h. Following this, mice were presented with NC in one hopper and HFD in the other for 24 h as a training period, then for 2 test days, with positions swapped every 24 h, and intake of each averaged.

### Taste bud isolation and RNA extraction

Circumvallate taste buds were isolated from mice after Lickometer testing and one week of wash out consuming NC and water ad libitum. Mouse tongues were freshly excised following euthanizing with CO_2_ and cervical dislocation. Circumvallate taste buds were extracted as previously^38^. Additionally, a piece of the epithelium posterior to the circumvallate papillae, termed the “non-taste” area was collected as a non-chemosensory control epithelial tissue, and processed in parallel. Quantitative real-time RT-PCR using Power SYBR Green PCR Master Mix (Applied Biosystems, Foster City, CA) was run on a QuantStudio 6 Flex Real-Time PCR System (Thermo Fisher Sci, Waltham, MA). Relative quantification was performed in triplicates using QuantStudio PCR Software, based on the 2^−ΔΔCt^ method. Beta-Actin was used as the endogenous housekeeping gene for normalization of genes of interest (see Supplemental Table 2).

### Postmortem tissue collection and staining

One hour prior to euthanizing, mice were injected with 5-HTP (5-Hydroxytryptophan, 2 mg/25 g body weight) to enhance immunofluorescence stain for serotonin positive taste cells. Mice were then euthanized with CO_2_ and cervical dislocation. Tongues were excised and rinsed in PBS. The circumvallate papilla was carefully isolated with a sterile razor and fixed in 4% PFA at 4 °C for one hour, cryoprotected in 30% sucrose overnight, and then embedded in OCT. Perigonadal fat pads were dissected and weighed. Livers were collected and immediately fixed in 10% neutral buffered formalin fixative for staining with oil red O. Liver sections were imaged using an Aperio CS2 at 40 × magnification (Leica, Wetzlar, Germany). Tongue tissue was sectioned at 10 microns and stained with hematoxylin and eosin (H&E) or antibodies for immunofluorescence (see Supplemental Table 3). Images were taken using an Olympus IX-71 microscope (Olympus Corp, Tokyo, Japan) with a Hamamatsu Orca Flash 4.0 camera (Hamamatsu Photonics, Hamamatsu, Japan). In order to avoid double counting single cells from taste buds in the circumvallate, every 8th section was used for quantification. Random taste buds from the left and right trenches of the circumvallate were selected, with a minimum of 10 taste buds per mouse (n = 4 per sex for each treatment). Slides were mounted using DAPI Fluoromount-G (4′,6-diamidino-2-phenylindole, Southern Biotech, Birmingham, AL). The anterior two-thirds of the mouse tongue was excised and fixed in 4% PFA for 24 h before staining with 1% methylene blue for 1 min, with tissue then rinsed with PBS, and imaged with an Olympus dissection scope (Olympus Corp, Tokyo, JP) with a Lumenera Infinity HD scope-mounted camera (Lumenera, Ottawa, CA), counted using ImageJ from a region of interest representing a 1 × 1 mm^2^.

### Statistical analysis

The effects of maternal HFD were analyzed using a two-way ANOVA with sex and maternal treatment groups as factors. When a significant interaction was identified, data were analyzed with post-hoc Tukey tests. Count data were analyzed with linear, or Poisson’s loglinear regression, depending on distributions. All analyses were carried out in GraphPad Prism 5.0, and using IBM SPSS 24 (IBM Corp, Armonk, NY), where results with *p* < 0.05 were considered statistically significant.

## Results

### Offspring phenotype appears similar between treatments

**Figure 2:**
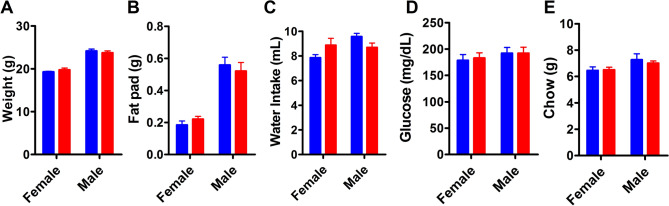
Metabolic parameters in maternal NC (blue) and HFD (red) offspring at 8–9 weeks of age. Values are expressed as mean ± SEM. (**A**) Mouse body weight. (**B**) Perigonadal fat pad mass. (**C**) Water intake over 48 h. (**D**) Blood glucose (**E**) Chow intake over 48 h. Data were analyzed by two-way ANOVA and post-hoc Tukey multiple comparisons test.

At 9 weeks of age the body weights for both sexes between the maternal treatments was comparable (Fig. 2A; n^NC^ = 10F, 8 M; n^HFD^ = 10F, 11 M; F_1,35_ = 1.313, *p*^diet^ = 0.2596). As well as having no impact on offspring’s body weight, maternal HFD exposure did not influence perigonadal fat pad weight, blood glucose levels, or baseline chow and water intake (Fig. 2B–E, all *p* > 0.05). Oil Red O staining measured hepatic lipid accumulation as a sign of any underlying disruption in fat metabolism between maternal treatment groups, and was unchanged between treatments (Supplemental Fig. 1A–C).

### Behavioral response to palatable food enhanced in HFD offspring

**Figure 3:**
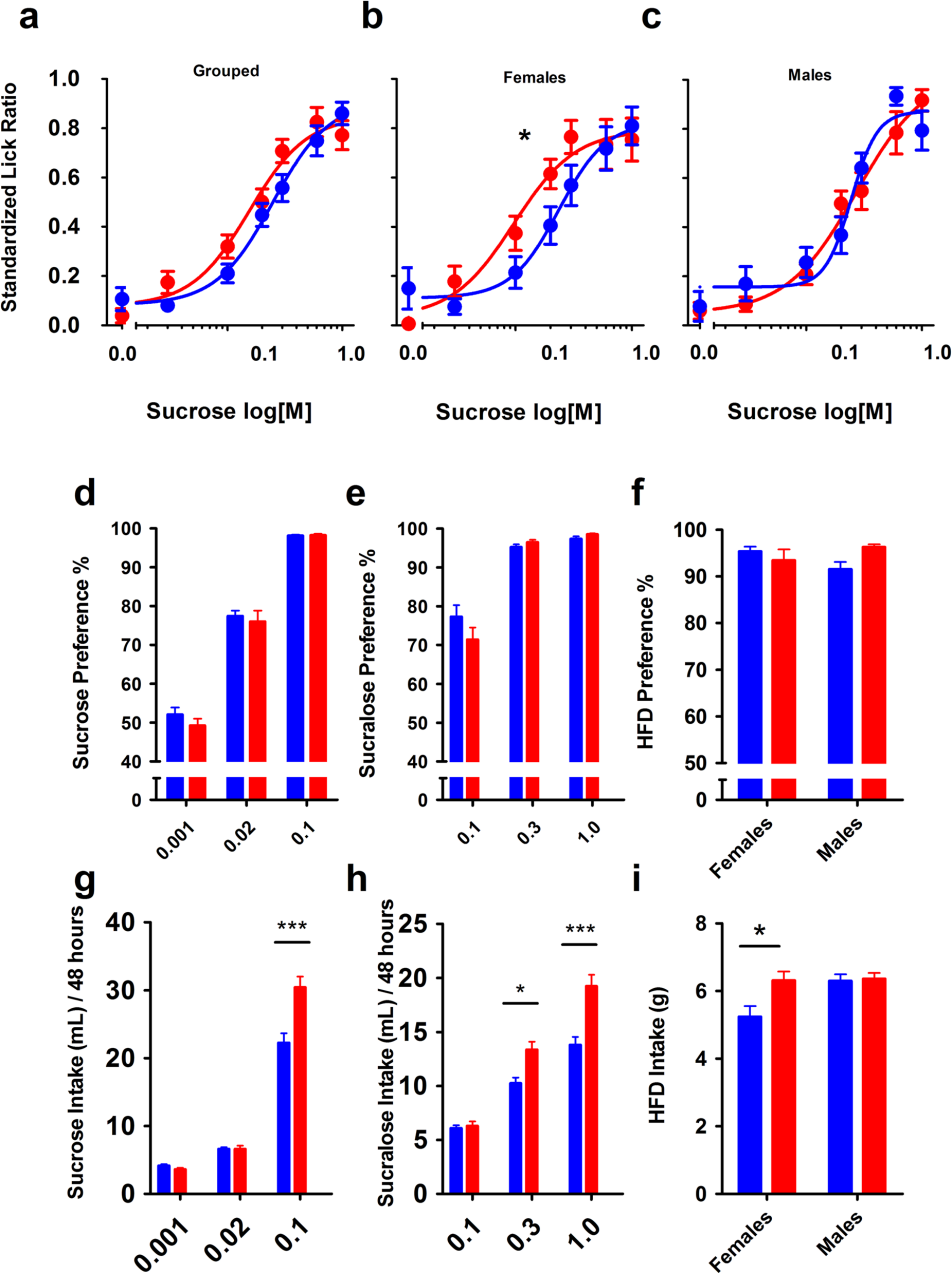
Behavioral responses of HFD offspring to appetitive stimuli differ from NC controls. Bars/points denote mean ± SEM. (**A**) Lickometer responses of offspring show a trend towards higher responsiveness to sucrose in maternal HFD offspring (red), compared to controls (blue), with female (**B**) offspring driving differences compared to males (**C**). Star represents statistical difference between curves. Preference (two-bottle, or two-diet) for sucrose (**D**, n = 9 M, 11F per treatment), sucralose (**E**, n = 9 M, 11F per treatment), and HFD (**F**, n = 7 HFD offspring, n = 8 controls) show similar patterns for HFD (red) offspring compared to controls (blue). 48 h intake patterns show enhanced consumption of sucrose (**G**), sucralose (**H**), and HFD (**I**) in offspring of HFD-fed dams (sample size as in **D**–**F**). Data were analyzed by two-way ANOVA and post-hoc Tukey multiple comparisons. Stars denote significance, where *p < 0.05; **p < 0.01; ***p < 0.001.

Brief-access sucrose responses were evaluated in the offspring (Fig. 3A–C). While treatments were not significantly different when grouped (n = 23; F_4,321_ = 2.008, *p* = 0.0932), differences between sex were evident. Female offspring of HFD-fed mice showed an enhanced licking response to sucrose compared to NC females (Fig. 3B; n = 12; F_4,167_ = 2.844, *p* = 0.026), while there remained no difference in males between treatments (Fig. 3C; n = 11; F_4,146_ = 0.903, *p* = 0.464).

Sweet preference and intake over 48 h were measured for both caloric (sucrose; Fig. 3D,G) and non-caloric (sucralose; Fig. 3E,H) solutions, in a separate group of naïve mice (n^NC^ = 20, n^HFD^ = 20). No significant differences in preference to sucrose (Fig. 3D) or sucralose (Fig. 3E) versus water among the two groups was evident, however, total intake of sweet solutions was enhanced in the maternal HFD treated offspring, with offspring of HFD treated mice consuming more sucrose (Fig. 3G; F_1,114_ = 11.95, *p* < 0.001) and sucralose (Fig. 3H; F_1,114_ = 28.27, *p* < 0.001) than respective controls. Elevated sucrose and sucralose intake while preference ratios remained similar between groups may have been due to elevated water intake along with sweet solutions, as preference data would take both into account, however this remains speculative. Preference for, and intake of high-fat diet was also evaluated in offspring (Fig. 3F,I). Female offspring of HFD-fed dams consumed significantly more HFD relative to control females (F_1,29_ = 5.684, *p* = 0.0239). Finally, HFD treatment applied either before conception but not while pregnant, or solely during gestation and lactation (thus in mice not obese before conception), did not elicit any change in sweet taste response in the offspring (Supplemental Fig. 2).

### Expression of taste signaling elements influenced by maternal diet

**Figure 4:**
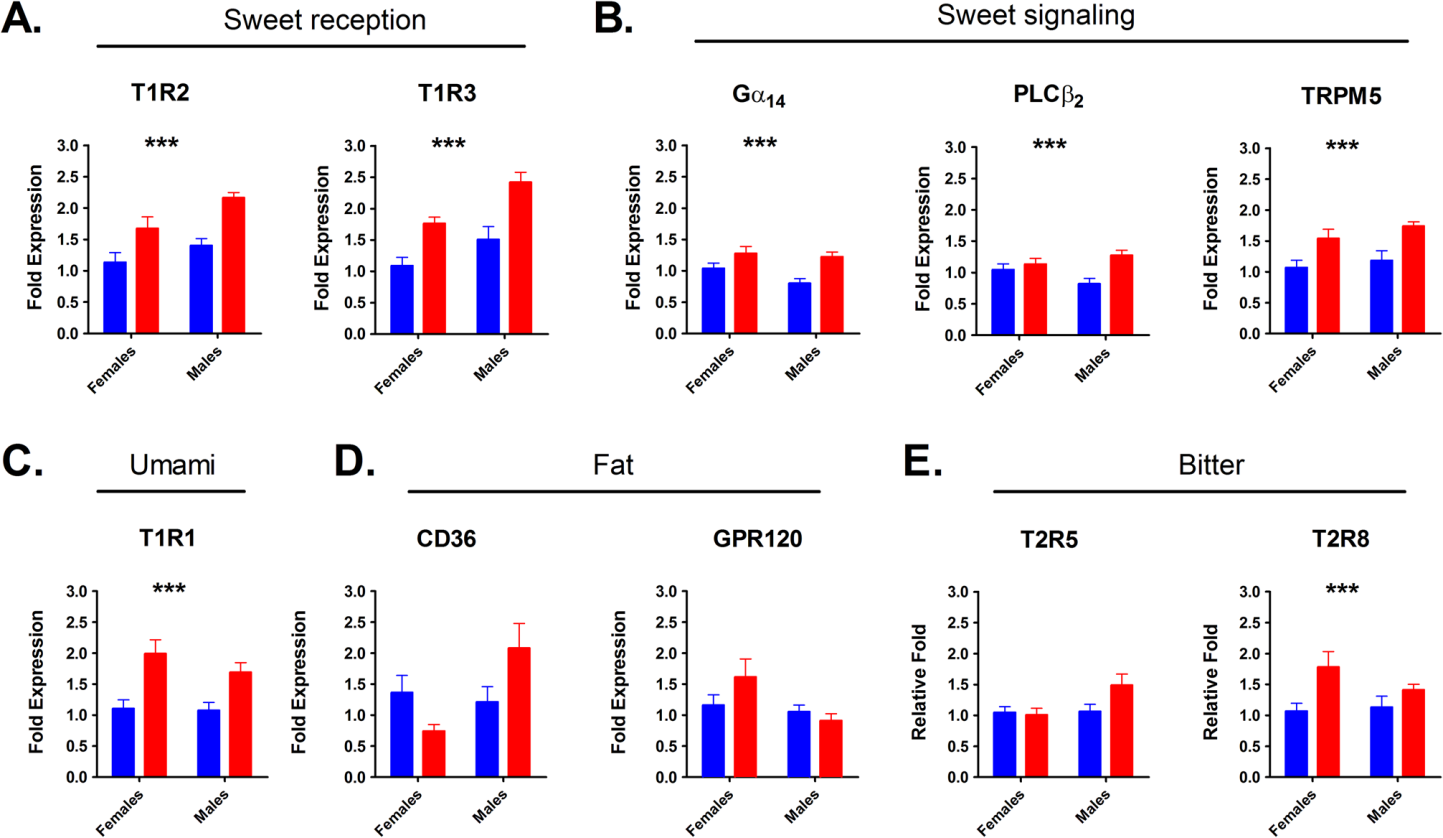
mRNA expression of taste bud signaling elements in NC (blue) and HFD (red) offspring. Bars denote mean ± SEM. Genes tested were sweet receptor subunits (**A**), sweet signaling components (**B**), umami (**C**), Fat (**D**) and bitter (**E**) taste receptors/detectors. Each biological sample (n = 4 mice per group) was run in triplicate. Data were analyzed by two-way ANOVA, with post-hoc Tukey multiple comparisons test. */**/*** signifies *p* < 0.05/0.01/0.001 for maternal diet main effect. Sex and interaction effects described in body.

Maternal diet influenced expression of all sweet receptor and sweet signaling genes tested (Fig. 4, n = 4 M and 4F each group, samples tested in triplicate). 2-way ANOVAs tested both main effects of maternal diet and sex, as well as interaction effect. For clarity, only significance of main effect of diet is displayed in Fig. 4. Testing revealed a clear effect of maternal diet on expression of mRNA encoding both parts of the sweet receptor heterodimer (Fig. 4A), T1R2 (*p*^diet^ < 0.001; *p*^sex^ = 0.001; *p*^interaction^ = 0.423) and T1R3 (*p*^diet^ < 0.001; *p*^sex^ = 0.001; *p*^interaction^ = 0.440). Similarly, members of the sweet signaling cascade (Fig. 4B) Gα-14 (Guanine nucleotide-binding protein subunit alpha-14) (*p*^diet^ = 0.001; *p*^sex^ = 0.011; *p*^interaction^ = 0.300), PLCβ2 (Phospholipase C beta-2) (*p*^diet^ = 0.004; *p*^sex^ = 0.657; *p*^interaction^ = 0.043), and TRPM5 (Transient receptor potential cation channel subfamily M member) (*p*^diet^ < 0.001; *p*^sex^ = 0.224; *p*^interaction^ = 0.735) were all significantly influenced by maternal dietary treatment, with the trend in each case being for higher RNA expression in offspring of maternal HFD-fed mice. Two-way ANOVAs showed a main effect of maternal treatment on gene expression of the umami receptor subunit T1R1 (*p*^diet^ < 0.001; *p*^sex^ = 0.323; *p*^interaction^ = 0.413) and bitter receptor T2R8 (*p*^diet^ = 0.005; *p*^sex^ = 0.376; *p*^interaction^ = 0.208), with a significant effect of sex and interaction between maternal treatment and sex detected for putative fat taste sensor CD36 (Fig. 4D; *p*^diet^ = 0.665; *p*^sex^ = 0.037; *p*^interaction^ = 0.001). No influence of maternal diet on expression of mRNA for putative fat receptor GPR120 (*p*^diet^ = 0.409; *p*^sex^ = 0.033; *p*^interaction^ = 0.113) or bitter receptor T2R5 (*p*^diet^ = 0.144; *p*^sex^ = 0.061; *p*^interaction^ = 0.080) was detected. All genes tested were taste-specific, and showed no modulation in non-taste tissue (Supplemental Fig. 3).

### Offspring display comparable taste bud morphology between treatment groups

**Figure 5:**
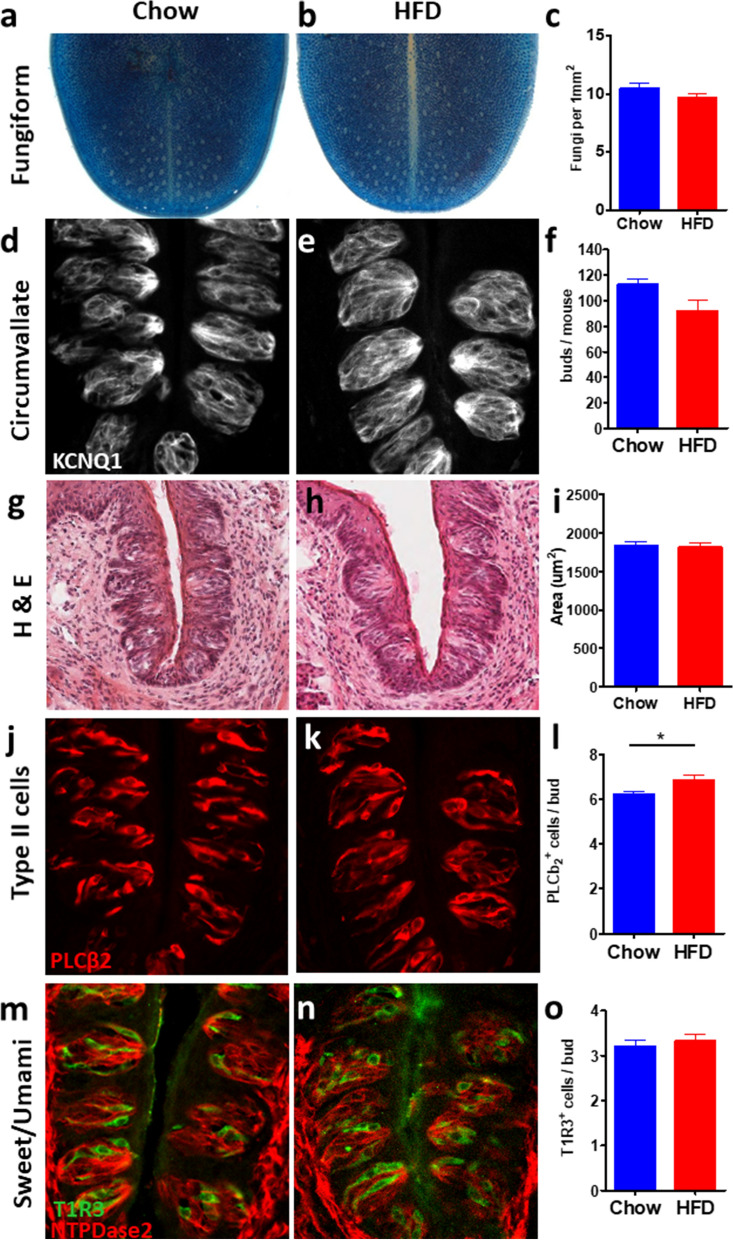
Taste bud morphology is similar in offspring of NC (blue) and HFD (red) fed dams. Bars denote mean ± SEM. (**A**) Image of anterior tongue of NC offspring stained with methylene blue to visualize fungiform papillae. (**B**) Anterior tongue of HFD offspring. (**C**) Fungiform density within 1 mm × 1 mm square in NC (n = 18) and HFD (n = 21) offspring. (**D**) Image of circumvallate taste buds of NC offspring. (**E**) HFD offspring. (**F**) quantification of taste buds/circumvallate papilla. (**G**) H&E stained CV papilla of NC offspring. (**H**) H&E image of HFD offspring papilla. (**I**) Taste bud size between treatment groups. (**J**) Immunofluorescent image of Type II taste cells in NC offspring. (**K**) Type II cells in HFD offspring. (**L**) Quantification of Type II cells. (**M**) Immunofluorescent image of sweet/bitter sensitive taste cells in NC offspring (green) with taste bud boundary highlighted (red). (**N**) Sweet/umami cells in HFD offspring. (**O**) Quantification of sweet/umami taste cells. Counting was performed from every 8 section of immunofluorescent stains, stars denote significance, where *p < 0.05; **p < 0.01; ***p < 0.001.

An alternative hypothesis leading to altered taste function or expression is a change in taste morphology. The fungiform papillae are easily accessible structures in the anterior tongue, housing one or more taste buds, and are often studied due to their easy access. No difference in fungiform density was detected between treatment groups (Fig. 5A–C; n^NC^ = 18, n^HFD^ = 21, *p* = 0.213). The circumvallate papillae in the posterior tongue house the highest density of taste buds in the mouth. For this reason, much taste research also concerns the circumvallate field. Circumvallate taste buds, stained with the general taste cell marker KCNQ1, also showed no difference in abundance between treatments (Fig. 5D–F; n = 4F, 4 M per group, *p* = 0.058). Taste bud size was quantified from ten circumvallate taste buds from each mouse (n = 80 total per treatment), and was also comparable between maternal treatment groups (Fig. 5G–I; *p* = 0.789). Finally, we tested whether the HFD group showed greater sweet response due to having more Type II taste cells (which includes sweet, umami, and bitter sensitive cells), or sweet/umami receptor-expressing cells compared to controls. Taste cells were analyzed from immunofluorescent images (n = 4F, 4 M per group) stained for PLCβ2 (Type II cells) or T1R3 (sweet/umami receptor subunit). Although analysis revealed more Type II taste cells in offspring of HFD-fed dams (Fig. 5J–l; *p* = 0.012), no significant difference in T1R3 positive cell abundance was detected between treatment groups (Fig. 5M–O; *p* = 0.547), suggesting any additional Type II cells may have arisen from bitter sensitive cells.

## Discussion

### Effects of maternal HFD on body weight, blood glucose, baseline water and chow intake, fat pads, and hepatic lipids

By week 9, no differences in body weight between maternal treatment groups was evident in our study (Fig. 2A), which corresponds to results from Vucetic et al.^36^. Additionally, there were no differences in perigonadal fat pad weights, blood glucose levels, or baseline water/chow intake between treatments for either sex (Fig. 2B–E). Other strains have shown contrasting results where offspring of HFD-fed dams had increased epididymal fat pad mass, and fatty livers, in Swiss dams^39^ and increased epididymal fat pads and altered liver histology in C3H/HeJ dams^40^, however in each of these measures, mice in our study were similar.

### Offspring of HFD-fed dams show increased avidity for palatable, high calorie stimuli

Female offspring of HFD-fed dams exhibited an enhanced licking response to sucrose compared to controls. This indicates that a lower concentration of sucrose induced the same level of licking response in the HFD group as would a higher concentration in controls. We also examined offspring of dams fed HFD solely during the pre-conception period, or solely during the gestation and lactation periods to determine if either time period could elicit the same increase in sweet responsiveness, but found no difference between groups (Supplementary Fig. 2). This suggests that maternal obesity and also the action of consuming HFD while pregnant underlies results, and is in line with studies by Treesukosol et al.^20^ in rats. Similarly, gestational exposure to sucralose in mice did not result in any change in sweet taste responsiveness, or taste receptor expression in the offspring^41^. Sex differences in the behavior of offspring from maternal feeding studies are common, with females noted in previous studies to be particularly prone to overconsumption^42,43^, possibly due to an alteration in VTA and nucleus accumbens mu-opioid receptor expression observed in female rats that seems absent in males^44^, or higher energy expenditure^45^ or altered plasma leptin levels^46^ in males. Maternal HFD treatment resulted in increased sucrose and sucralose intake in offspring of both sexes compared to controls, without any direct exposure to HFD, sucrose or sucralose. Interestingly in humans, a decrease in sweet taste response has been linked with a desire for greater concentrations of sucrose^47^, suggesting that an acute versus chronic change in taste may lead to contrasting outcomes. Although offspring had never been directly exposed to HFD, the female offspring of HFD fed dams consumed more HFD than controls (Fig. 3I), suggesting enhanced sweet taste may contribute to a propensity to over-consume palatable foods in general, other taste machinery may be altered as suggested by results in Fig. 4, or that the formulation of HFD presents as sweeter than regular chow; all likely outcomes. Other studies have also shown higher HFD intake in offspring of maternal HFD treatment^19–21^, and that a maternal “junk food” diet^42^, causes female rats to increase intake of fats, where males do not.

### Maternal obesity alters gene expression in the taste buds of the offspring

Sweet taste usually implies calories, and thus can induce an appetitive response to promote ingestion^48^. We examined gene expression of mRNA for sweet receptor subunits T1R2 and T1R3, as well as several additional taste signaling elements.

In offspring of HFD fed mice, we found increased T1R2 and T1R3 sweet receptor mRNA expression, as well as for other components of the sweet signaling pathway (Fig. 4). Although the functional consequences of increased sweet receptor mRNA expression are unknown, genetic deletion of T1R3 in mice results in reduced consumption of carbohydrates and fats^49,50^. Additionally, rats fed HFD for 6 weeks showed decreased T1R3 expression, which was associated with lower intake and preference ratios for saccharin solutions^51^. While this may represent one mechanism for a change in taste responsiveness in offspring of HFD fed dams, several other explanations should not be discounted. Among these, an alteration in food reward, changes to second messenger cascades beyond those assayed in our experiments, or a varied pattern of taste innervation in the progeny of HFD fed vs control dams would all be rational. An additional mechanism that could potentially alter responsiveness to sweet and fat stimuli is an altered balance of cell-types within the taste buds of offspring of obese mice, with more PLCβ2 positive cells in our results. PLCβ2 marks type 2 taste cells, and thus the cells that are responsible for the detection of sweet, bitter and umami tastes. The free fatty acid G-protein coupled receptor GPR40 (Ffar1), a proposed fat taste receptor, is located at least primarily in Type 2 taste cells^52^, with another, CD36, colocalizing in taste buds with α-gustducin^53^, another well-accepted Type 2 marker. Nonetheless, the upregulated cell population did not seem to be sweet-sensitive, with T1R3 cells unchanged between groups, suggesting that the expanded population may have been primarily bitter-sensitive. The reduction in CD36 observed in female offspring of HFD fed mice, accompanied by an increase in GPR120 parallels the findings of Ozdener et al. in cultured mouse and human taste cells chronically exposed to linoleic acid^54^. Another potential mechanism for differences between offspring groups would be due to differing metabolite profiles arising from the breakdown of fatty acids in the diets (for more on fat taste signaling see reviews^55,56^). The balance of fatty acids between control and high fat diets varied in our setup as well as simply the fat percentage^34^, with saturated and monounsaturated fatty acids in particular increased in the HFD group, which would have divergent effects on metabolism in the dam and likely also the offspring. Finally, another likely source of regulation in the offspring of HFD-fed dams would be central reward circuits governing intake. We would not rule out additional regulation of such circuits, as shown in the offspring of rats fed a junk-food diet by Ong and Muhlhausler^57^, occurring alongside taste receptor regulation, unbalancing feeding behavior in these animals.

This work provides evidence that the maternal environment can result in long-term programming effects on the taste system. Permanent alterations and stable long-term repression of some genes maintained through cell division, as suggested by our results, could be the result of DNA methylation or histone modification^58^, as a maternal HFD has been shown to influence epigenetic machinery^59^. Increased preference for fat and sucrose in offspring of maternal HFD fed animals is associated with global and gene specific decreases in DNA methylation in the offspring brain^36^. Thus, maternal obesity may mediate long-term changes in gene expression in the offspring, including expression of taste receptors, via epigenetic regulation. While the specific mechanism remains speculative at this point, our results introduce taste to the growing list of metabolic alterations arising from fetal programming, and add to the growing evidence that the taste bud plays a role in the etiology of obesity.

## Conclusions

Overall, this study is the first to demonstrate that maternal exposure to HFD during the perinatal period leads to long-term consequences for taste signaling components, as well as feeding behavior. Offspring of dams fed a high-fat diet exhibited enhanced licking responses for sucrose solutions, as well as increased intake of palatable stimuli (sucrose, sucralose and high fat diet), while remaining equally responsive to neutral stimuli (regular chow and water). From a public health standpoint, improving our knowledge of prenatal and early postnatal factors that program obesity in the offspring may provide insight into therapeutic targets to combat the obesity epidemic, a disease easier to prevent than to cure.

## Supplementary information

Supplementary Information.

## References

[1] Kelly T, Yang W, Chen CS, Reynolds K, He J (2008). Global burden of obesity in 2005 and projections to 2030. Int. J. Obes..

[2] Adair LS (2008). Child and adolescent obesity: epidemiology and developmental perspectives. Physiol. Behav..

[3] Vahratian A (2009). Prevalence of overweight and obesity among women of childbearing age: results from the 2002 National Survey of Family Growth. Matern. Child Health J..

[4] Catalano PM, Presley L, Minium J, Hauguel-de MS (2009). Fetuses of obese mothers develop insulin resistance in utero. Diabetes Care.

[5] Barker DJ (1994). Mothers, babies, and disease in later life.

[6] Barker DJ (1995). Fetal origins of coronary heart disease. BMJ.

[7] Drake AJ, Reynolds RM (2010). Impact of maternal obesity on offspring obesity and cardiometabolic disease risk. Reproduction.

[8] Alfaradhi MZ, Ozanne SE (2011). Developmental programming in response to maternal overnutrition. Front. Genet..

[9] Li M, Sloboda DM, Vickers MH (2011). Maternal obesity and developmental programming of metabolic disorders in offspring: evidence from animal models. Exp. Diabetes Res..

[10] Kaufman A, Choo E, Koh A, Dando R (2018). Inflammation arising from obesity reduces taste bud abundance and inhibits renewal. PLoS Biol..

[11] Kaufman A, Kim J, Noel C, Dando R (2019). Taste loss with obesity in mice and men. Int. J. Obes..

[12] Noel CA, Cassano PA, Dando R (2017). College-aged males experience attenuated sweet and salty taste with modest weight gain. J. Nutr..

[13] Benkalfat NB, Merzouk H, Bouanane S, Merzouk SA, Bellenger J, Gresti J, Tessier C, Narce M (2011). Altered adipose tissue metabolism in offspring of dietary obese rat dams. Clin. Sci..

[14] Howie GJ, Sloboda DM, Kamal T, Vickers MH (2009). Maternal nutritional history predicts obesity in adult offspring independent of postnatal diet. J. Physiol..

[15] Page KC, Malik RE, Ripple JA, Anday EK (2009). Maternal and postweaning diet interaction alters hypothalamic gene expression and modulates response to a high-fat diet in male offspring. Am. J. Physiol. Reg. Integr..

[16] Dietrich MO, Bober J, Ferreira JG, Tellez LA, Mineur YS, Souza DO, Gao XB, Picciotto MR, Araujo I, Liu ZW, Horvath TL (2019). AgRP neurons regulate development of dopamine neuronal plasticity and nonfood-associated behaviors. Nat. Neurosci..

[17] Penfold NC, Ozanne SE (2015). Developmental programming by maternal obesity in 2015: Outcomes, mechanisms, and potential interventions. Horm. Behav..

[18] Choo E, Dando R (2017). The impact of pregnancy on taste function. Chem. Senses.

[19] Tamashiro KLK, Terrillion CE, Hyun J, Koenig JI, Moran TH (2009). Prenatal stress or high-fat diet increases susceptibility to diet-induced obesity in rat offspring. Diabetes.

[20] Treesukosol Y, Sun B, Moghadam AA, Liang NC, Tamashiro KL, Moran TH (2014). Maternal high-fat diet during pregnancy and lactation reduces the appetitive behavioral component in female offspring tested in a brief-access taste procedure. Am. J. Physiol. Regul. Integr. Comp. Physiol..

[21] Bayol SA, Farrington SJ, Stickland NC (2007). A maternal ‘junk food’ diet in pregnancy and lactation promotes an exacerbated taste for ‘junk food’ and a greater propensity for obesity in rat offspring. Br. J. Nutr..

[22] Teegarden SL, Scott AN, Bale TL (2009). Early life exposure to a high fat diet promotes long-term changes in dietary preferences and central reward signaling. Neuroscience.

[23] Naef L, Moquin L, Bo GD, Giros B, Gratton A, Walker CD (2011). Maternal high-fat intake alters presynaptic regulation of dopamine in the nucleus accumbens and increases motivation for fat rewards in the offspring. Neuroscience.

[24] Lanfer A, Knof K, Barba G, Veidebaum T, Papoutsou S (2012). Taste preferences in association with dietary habits and weight status in European children: results from the IDEFICS study. Int. J. Obes..

[25] Rivera HM, Kievit P, Kirigiti MA, Bauman LA, Baquero K, Blundell P, Dean TA (2015). Maternal high-fat diet and obesity impact palatable food intake and dopamine signaling in nonhuman primate offspring. Obesity.

[26] Kern DL, McPhee L, Fisher J, Johnson S, Birch LL (1993). The postingestive consequences of fat condition preferences for flavors associated with high dietary fat. Physiol. Behav..

[27] Liem DG (2002). Mennella JA (2002) Sweet and sour preferences during childhood: role of early experiences. Dev. Psychobiol..

[28] Mennella JA, Beauchamp GK (2002). Flavor experiences during formula feeding are related to preferences during childhood. Early Hum. Dev..

[29] Mennella JA (2014). Ontogeny of taste preferences. Basic biology and implications for health. Am. J. Clin. Nutr..

[30] Beauchamp GK, Mennella JA (2009). Early flavor learning and its impact on alter feeding behavior. J. Pedr. Gastroenterol. Nutr. J..

[31] Beauchamp GK, Mennella JA (2011). Flavor perception in human infants: development and functional significance. Digestion.

[32] Mennella JA, Jagnow CP, Beauchamp GK (2001). Prenatal and postnatal flavor learning by human infants. Pediatrics.

[33] Rando OJ, Simmons RA (2015). I’m eating for two: Parental dietary effects on offspring metabolism. Cell.

[34] Ferramosca A, Conte A, Burri L, Berge K, De Nuccio F, Giudetti AM, Zara V (2012). A krill oil supplemented diet suppresses hepatic steatosis in high-fat fed rats. PLoS ONE.

[35] Glendinning JI, Gresack J, Spector AC (2002). A high-throughput screening procedure for identifying mice with aberrant taste and oromotor function. Chem. Senses.

[36] Vucetic Z, Kimmel J, Totoki K, Hollenbeck E, Reyes TM (2010). Maternal high-fat diet alters methylation and gene expression of dopamine and opioid-related genes. Endocrinology.

[37] Carlin JL, George R, Reyes TM (2013). Methyl donor supplementation blocks the adverse effects of maternal high fat diet on offspring physiology. PLoS ONE.

[38] Dando R, Dvoryanchikov G, Pereira E, Chaudhari N, Roper SD (2012). Adenosine enhances sweet taste through A2B receptors in the taste bud. J. Neurosci..

[39] Ashino NG, Saito KN, Souza FD, Nakutz FS, Roman EA, Velloso LA, Torsoni AS, Torsoni MA (2012). Maternal high-fat feeding through pregnancy and lactation predisposes mouse offspring to molecular insulin resistance and fatty liver. J. Nutr. Biochem..

[40] Walter I, Klaus S (2014). Maternal high-fat diet consumption impairs exercise performance in offspring. J. Nutr. Sci..

[41] Choo E, Dando R (2018). No detriment in taste response or expression in offspring of mice fed representative levels of sucrose or non-caloric sucralose while pregnant. Physiol. Behav..

[42] Gugusheff JR, Ong ZY, Muhlhausler BS (2013). A maternal “junk-food” diet reduces sensitivity to the opioid antagonist naloxone in offspring postweaning. FASEB J..

[43] Bellinger L, Lilley C, Langley-Evans SC (2004). Prenatal exposure to a maternal low-protein diet programmes a preference for high-fat foods in the young adult rat. Br. J. Nut..

[44] Gugusheff JR, Bae SE, Rao A, Clarke IJ, Poston L, Taylor PD, Coen CW, Muhlhausler BS (2016). Sex and age-dependent effects of a maternal junk food diet on the mu-opioid receptor in rat offspring. Behav. Brain Res..

[45] Dias-Rocha CP, Almeida MM, Santana EM, Costa JC, Franco JG, Pazos-Moura CC, Trevenzoli IH (2018). Maternal high-fat diet induces sex-specific endocannabinoid system changes in newborn rats and programs adiposity, energy expenditure and food preference in adulthood. J. Nutr. Biochem..

[46] Sánchez J, Priego T, García AP, Llopis M, Palou M, Picó C, Palou A (2012). Maternal supplementation with an excess of different fat sources during pregnancy and lactation differentially affects feeding behavior in offspring: putative role of the leptin system. Mol. Nutr. Food Res..

[47] Noel CA, Sugrue M, Dando R (2017). Participants with pharmacologically impaired taste function seek out more intense, higher calorie stimuli. Appetite.

[48] Anderson GH (1995). Sugars, sweetness, and food intake. Am. J. Clin. Nutr..

[49] Glendinning JI, Gillman J, Zamer H (2012). The role of T1r3 and Trpm5 in carbohydrate-induced obesity in mice. Physiol. Behav..

[50] Larsson MH, Hakansson P, Jansen FP (2015). Ablation of TRPM5 in mice results in reduced body weight gain and improved glucose tolerance and protects from excessive consumption of sweet palatable food when fed high caloric diets. PLoS ONE.

[51] Chen K, Yan J, Suo Y, Li J, Wang Q, Lv B (2010). Nutritional status alters saccharin intake and sweet receptor mRNA expression in rat taste buds. Brain Res..

[52] Cartoni C, Yasumatsu K, Ohkuri T, Shigemura N, Yoshida R, Godinot N, Le Coutre J, Ninomiya Y, Damak S (2010). Taste preference for fatty acids is mediated by GPR40 and GPR120. J. Neurosci..

[53] Laugerette F, Passilly-Degrace P, Patris B, Niot I, Febbraio M, Montmayeur JP, Besnard P (2005). CD36 involvement in orosensory detection of dietary lipids, spontaneous fat preference, and digestive secretions. J. Clin. Invest..

[54] Ozdener MH, Subramaniam S, Sundaresan S, Sery O, Hashimoto T, Asakawa Y, Besnard P, Abumrad NA, Khan NA (2014). CD36-and GPR120-mediated Ca^2+^ signaling in human taste bud cells mediates differential responses to fatty acids and is altered in obese mice. Gastroenterology.

[55] Gilbertson TA, Khan NA (2014). Cell signaling mechanisms of oro-gustatory detection of dietary fat: advances and challenges. Prog. Lipid Res..

[56] Besnard P, Passilly-Degrace P, Khan NA (2016). Taste of fat: a sixth taste modality?. Physiol. Rev..

[57] Ong ZY, Muhlhausler BS (2011). Maternal “junk-food” feeding of rat dams alters food choices and development of the mesolimbic reward pathway in the offspring. FASEB J..

[58] Cedar H, Bergman Y (2009). Linking DNA methylation and histone modification: patterns and paradigms. Nat. Rev. Genet..

[59] Panchenko PE, Voisin S, Jouin M, Jouneau L, Prézelin A, Lecoutre S, Breton C, Jammes H, Junien C, Gabory A (2016). Expression of epigenetic machinery genes is sensitive to maternal obesity and weight loss in relation to fetal growth in mice. Clin. Epigenet..

