# Supplementary material for: Offspring of obese mice display enhanced intake and sensitivity for palatable stimuli, with altered expression of taste signaling elements
Source: Sci Rep. 2020 Jul 29;10:12776. doi: 10.1038/s41598-020-68216-7 (PMC7391633; doi:10.1038/s41598-020-68216-7)
Supplement: Supplementary file 1 — Supplementary Information. [file 41598_2020_68216_MOESM1_ESM.docx]

**Offspring of obese mice display enhanced intake and sensitivity for palatable stimuli, with altered expression of taste signaling elements**

Ezen Choo^1^, Lauren Wong^2^, Patricia Chau^3^, Jennifer Bushnell^4^, & Robin Dando^5^

^1^Biomedical & Biological Sciences, College of Veterinary Medicine, Cornell University, Ithaca, NY 14853, ^2^College of Arts and Sciences, Cornell University, Ithaca, NY 14853, ^3^Division of Nutritional Sciences, Cornell University, Ithaca, NY 14853, ^4^College of Engineering, Cornell University, Ithaca, NY 14853, ^5^Department of Food Science, Cornell University, Ithaca, NY 14853

**Supplemental Tables**

**Supplemental Table 1**

|  |  | **NC** |  |  |  | **HFD^56^** |  |
| --- | --- | --- | --- | --- | --- | --- | --- |
| **Nutrients** | g | Kcal | %Kcal |  | g | Kcal | %Kcal |
| Fat | 6.2 | 55.8 | 18.0 |  | 20.4 | 315.4 | 58.4 |
| Protein | 18.6 | 74.4 | 24.0 |  | 36.1 | 81.0 | 15.0 |
| Carbohydrate | 44.2 | 179.8 | 58.0 |  | 35.2 | 143.6 | 26.6 |
| Total |  | 310 | 100 |  |  | 540 | 100 |
| Kcal/g |  | 3.1 |  |  |  | 5.4 |  |

**Table 1. Content of fat, protein, and carbohydrate in the normal control diet (NC) and high-fat diet (HFD), per 100g.**

**Supplemental Table 2**

| Protein | Gene | Forward 5'-3' | Reverse 5'-3' | Product size (bp) |
| --- | --- | --- | --- | --- |
| β-actin | *Actb* | caccctgtgctgctcacc | gcacgatttccctctcag | 328 |
| T1R2 | *Tas1r2* | aagcatcgcctcctactcc | ggctggcaactcttagaacac | 114 |
| T1R3 | *Tas1r3* | gaagcatccagatgacttca | gggaacagaaggacactgag | 283 |
| Gα14 | *Gna14* | attagctacttcccagagtacaca | gctcagatcaccctctgtct | 256 |
| PLCβ2 | *Plcb2* | gagcaaatcgccaagatgat | ccttgtctgtggtgaccttg | 163 |
| TrpM5 | *Trpm5* | gtctggaatcacaggccaac | gttgatgtgccccaaaaact | 234 |
| T1R1 | *Tas1r1* | ctggaatggacctgaatggac | agcagcagtggtgggaac | 185 |
| CD36 | *Cd36* | ggccaagctattgcgacatg | ccgaacacagcgtagatagac | 124 |
| GPR120 | *Gpr120* | ctggggctcatctttgtcgt | acgacgagcactagagggat | 155 |
| T2R5 | *Tas2r105* | gaatcatagaaacaggacctcg | ctttacaaaggcttgctttagc | 406 |
| T2R8 | *Tas2r108* | ttctgatttcagccctcacc | ccaaaagctggtcctgtttc | 245 |

**Table 2. Genes of interest and their corresponding primer sequences.**

**Supplemental Table 3**

| Antigen | Host | Vender | Dilution |
| --- | --- | --- | --- |
| NTPDase2 | rabbit | J. Sévigny at Université Laval, Quebec | 1:1000 |
| PLCβ2 | rabbit | Santa Cruz Biotechnology | 1:1000 |
| IP3R3 | goat | Santa Cruz Biotechnology | 1:1000 |
| T1R2 | goat | Santa Cruz Biotechnology | 1:200 |
| T1R3 | goat | Santa Cruz Biotechnology | 1:1000 |
| KCNQ1 | goat | Santa Cruz Biotechnology | 1:1000 |
| 5HT | rat | Millipore | 1:1000 |
| Gustducin | rabbit | Santa Cruz Biotechnology | 1:1000 |
| Ki67 | rabbit | Thermo | 1:1000 |
| Sox2 | goat | Santa Cruz Biotechnology | 1:1000 |

**Table 3. Primary antibodies used for immunofluorescence analysis**

**Supplemental Figures**

**Supplemental Figure 1.** A. Hepatic lipid content quantified from images analyzed using color deconvolution, n=6 per group, 3 sections per mouse. B, C. Liver sections from chow (G) and HFD (H) offspring, stained with Oil Red O, at 40x magnification, bar = 200um. Bars denote mean ± SEM.





**Supplemental Figure 2.** Intermediate maternal treatment groups. (A) Maternal chow-HFD, excess fat consumption only during gestation/ lactation, i.e. gestational weight gain, green, n = 20 vs 19 controls, blue and (B) maternal HFD-chow, high-fat diet consumption only prior to conception, purple n = 22 vs 19 controls, blue) showed no alteration in sweet taste responses in female or male offspring. Points denote mean ± SEM.

**Supplemental Figure 3.** Non-taste samples were processed and analyzed in parallel as a negative control for taste samples in females (A) and males (B). All genes examined were taste specific, so we saw little to no expression in all non-taste samples. Bars denote mean ± SEM.
